# Angiogenic Potential of VEGF Mimetic Peptides for the Biofunctionalization of Collagen/Hydroxyapatite Composites

**DOI:** 10.3390/biom11101538

**Published:** 2021-10-19

**Authors:** Suya Wang, Felix Umrath, Wanjing Cen, Siegmar Reinert, Dorothea Alexander

**Affiliations:** Department of Oral and Maxillofacial Surgery, University Hospital Tübingen, Osianderstr. 2–8, 72076 Tübingen, Germany; suya.wang@student.uni-tuebingen.de (S.W.); felix.umrath@med.uni-tuebingen.de (F.U.); cenwanjingwj@gmail.com (W.C.); siegmar.reinert@med.uni-tuebingen.de (S.R.)

**Keywords:** collagen/hydroxyapatite composites, tissue engineering, vascular endothelial growth factor (VEGF), VEGF mimicry peptides, human umbilical vein endothelial cells (HUVECs), angiogenesis

## Abstract

Currently, the focus on bioinspired concepts for the development of tissue engineering constructs is increasing. For this purpose, the combination of collagen (Coll) and hydroxyapatite (HA) comes closest to the natural composition of the bone. In order to confer angiogenic properties to the scaffold material, vascular endothelial growth factor (VEGF) is frequently used. In the present study, we used a VEGF mimetic peptide (QK) and a modified QK-peptide with a poly-glutamic acid tag (E7-QK) to enhance binding to HA, and analyzed in detail binding efficiency and angiogenic properties. We detected a significantly higher binding efficiency of E7-QK peptides to hydroxyapatite particles compared to the unmodified QK-peptide. Tube formation assays revealed similar angiogenic functions of E7-QK peptide (1µM) as induced by the entire VEGF protein. Analyses of gene expression of angiogenic factors and their receptors (*FLT-1, KDR, HGF, MET, IL-8, HIF-1α, MMP-1, IGFBP-1, IGFBP-2, VCAM-1*, and *ANGPT-1*) showed higher expression levels in HUVECs cultured in the presence of 1 µM E7-QK and VEGF compared to those detected in the negative control group without any angiogenic stimuli. In contrast, the expression of the anti-angiogenic gene TIMP-1 showed lower mRNA levels in HUVECs cultured with E7-QK and VEGF. Sprouting assays with HUVEC spheroids within Coll/HA/E7-QK scaffolds showed significantly longer sprouts compared to those induced within Coll/HA/QK or Coll/HA scaffolds. Our results demonstrate a significantly better functionality of the E7-QK peptide, electrostatically bound to hydroxyapatite particles compared to that of unmodified QK peptide. We conclude that the used E7-QK peptide represents an excellently suited biomolecule for the generation of collagen/hydroxyapatite composites with angiogenic properties.

## 1. Introduction

Regeneration of critical size bone defects in oral and maxillofacial surgery requires challenging reconstructive surgery techniques [1]. In the clinical practice, the most common surgical procedures for bone regeneration are bone grafts, including autologous, allogenic and xenogenic bone grafts or prosthetic biomaterial implants [2]. To date, the gold standard for bone regeneration is still the use of autologous bone tissue. However, these approaches are limited by insufficient neovascularization, and integration within the host tissue, or by immune rejection in the case of allogenic/xenogenic grafts [3,4,5]. As oxygen and nutrient supply is critical for the survival of a graft, new materials have to be developed exhibiting both osteoinductive and angiogenic properties.

In order to avoid the creation of a second bone defect caused by autologous bone grafting, bone tissue engineering is a promising alternative. This therapeutic procedure benefits from the regenerative capacity of the human body by the application of adult stem cells in combination with optimized synthetic materials [6]. Calcium phosphate (CaP) biomaterials are often chosen as scaffolds to combine with stem cells, organic components, or with other materials such as polymers to induce new bone formation at the defect site [7]. Hydroxyapatite (HA) is the main inorganic component of the natural bone, and it exhibits excellent biocompatibility with soft tissues. Composite scaffolds containing HA and polymers [8,9,10,11,12] or HA and collagen have been developed [13,14,15,16,17] to mimic the natural bone composition. Type-I collagen represents the main organic component of the bone, and it initiates and orientates the growth of carbonated apatite minerals [18]. In a recent comparative study, nano-hydroxyapatite/collagen composites showed higher Young’s moduli and higher induction of late osteogenesis marker expression compared to natural bone ceramic/collagen scaffolds [19]. Further, the combination of collagen with nano-hydroxyapatite demonstrated excellent mechanical and biological properties [20].

The vascularization of bone grafts is a necessity for significant bone ingrowth. The process of angiogenesis runs in parallel to the process of osteogenesis, providing oxygen and nutrients to osteogenic and endothelial cells, thus ensuring their survival and subsequent differentiation [21]. Therefore, in addition to required osteogenic growth factors, sustained delivery of angiogenic growth factors such as vascular endothelial growth factor (VEGF) plays an important role in bone regeneration [22]. However, some studies have demonstrated that the use of growth factor proteins has several inherent disadvantages, such as immunogenicity, lower stability, and loss of bioactivity. Therefore, the use of short peptides containing binding sites for the respective receptors instead of whole recombinant proteins, shall be pursued to avoid side effects and to minimize costs [23]. The QK peptide is a VEGF-mimetic angiogenic peptide which was shown to improve significantly the migration and proliferation of endothelial cells and to promote neovascularization [24]. It is a synthetic 15 amino acid peptide containing the 17–25 alpha region of the VEGF165 protein [25]. The QK peptide binds to the VEGFR-1 and 2 receptors, and further activates the same angiogenic response as VEGF does [26].

Multiple strategies have been identified and tested for the functionalization of implant materials with bioactive factors [27,28,29,30]. Several studies demonstrated that glutamic acid residues within proteins are essential for the specific interaction with the crystalline structure of the main inorganic component of the bone, hydroxyapatite [31,32]. In a previous work, we used a modified BMP-2 mimetic peptide, containing a polyglutamic acid residue (E7-tag), for the incorporation into a collagen/HA composite. The included E7-tag confers accumulated negative charges and thereby a high binding affinity to positively charged hydroxyapatite surfaces [33]. This method for immobilization has the big advantage that it needs no chemical reactions or organic solvents for cross-linking, which can influence biomolecule activity and/or physicochemical integrity of the biomaterial surface of scaffolds [20]. In order to increase attraction and intrusion of host endothelial cells, we aim to include also angiogenic factors into the in vitro developed scaffolds. Therefore, the aim of the current study is to analyze a VEGF-mimetic (QK) peptide containing an E7-tag (polyglutamic acid residue) in terms of binding affinity to hydroxyapatite (HA) as well as in terms of its functionality concerning angiogenic properties in the 2D culture and within a 3D collagen/HA scaffold.

## 2. Materials and Methods

### 2.1. Preparation of Collagen/Hydroxyapatite Scaffolds

Collagen/hydroxyapatite composites were prepared according to a previously established protocol [33]. Briefly, the required amount of hydroxyapatite nanoparticles (10 µm powder, Fluidinova, Moreira da Maia, Portugal) was weighted, washed with TBS (tris buffered saline, Biolegend), and then equilibrated in TBS overnight. Thereafter, the TBS/HA solution was centrifuged for 20 min at 3000 rpm. TBS supernatant was discarded and a VEGF-mimicry peptide solution (200 µg peptide in 400 µL TBS (molar concentration: QK—0.52 mM; E7-QK—0.35 mM) per 10 mg HA) was added and incubated with HA for 2 h while rotating (15 rpm). The respective volume of the bovine type I collagen solution (HA: Coll = 2:1) (Advanced Biomatrix, San Diego, CA, USA) was added and then put into an incubator for polymerization at 37 °C for 30 min.

### 2.2. VEGF Mimetic Peptides

All peptides in this study were customized and synthesized by Biomatik (Wilmington, DE, USA). Three different peptides were used: the unmodified VEGF-derived QK peptide (QK-peptide: KLTWQELQLKYKGIGGG, MW = 1919.24 g/mol), E7-tag (poly-glutamic acid) modified QK peptide for stronger binding to hydroxyapatite (E7-QK peptide: EEEEEEEKLTWQELYQLKYKGI, MW = 2823.03 g/mol), and E7 & TAMRA modified QK peptide for the visualization within the 3D coll/HA scaffold (E7-QK-TAMRA peptide: Ac-EEEEEEEKLTWQELYQLKYKGI-Lys (TAMRA), MW = 3397.69 g/mol).

### 2.3. Quantification of the Binding Efficiencies of Different Peptides to HA

Electrostatic binding of the QK peptides to the positively charged Ca^2+^ ions of the hydroxyapatite (HA) lattice, occurs through negatively charged amino acid residues and is conferred by the inserted glutamic acid residues (E7-tag) or the TAMRA tag (fluorochrome labeling for visualization). In order to quantify the binding efficiencies of the different unmodified and modified peptides to HA, the concentration of unbound peptide after 2 h of incubation with a solution containing HA particles was measured. Briefly, 10 mg of the hydroxyapatite powder (Fluidinova, Moreira da Maia, Portugal) was weighted and washed once with TBS buffer (0.15 M NaCl/50 mM Tris/HCl, pH 7.4). After equilibration in TBS overnight and centrifugation, peptide solutions of 0.25 mM/10 mg HA (total volume 400 µL) were incubated for 2 h at room temperature under continuous rotation (15 rpm). Peptide concentrations were determined in the supernatants of the solutions before and after incubation with HA particles using the Micro BCA^TM^ protein assay kit (Pierce, Thermo Scientific, Waltham, MA, USA) according to the manufacturer’s instructions.

### 2.4. Visualization of E7-QK Peptide Incorporated into the Collagen/Hydroxyapatite Scaffolds

The TAMRA-labeled E7-QK peptide was used in order to visualize peptide binding to HA and retention within the collagen/hydroxyapatite scaffold. First, 10 mg of the hydroxyapatite powder (Fluidinova, Moreira da Maia, Portugal) was weighted and washed once with TBS buffer (0.15 M NaCl/50 mM Tris/HCl, pH 7.4). After equilibration in TBS buffer overnight and centrifugation, 200 µg peptide in 400 µL TBS (E7-QK-TAMRA—0.15 mM) were incubated with 10 mg HA for 2 h at room temperature under continuous rotation (15 rpm) and protected from light. One milliliter of bovine type I collagen solution (Advanced Biomatrix, San Diego, CA, USA) was added, mixed completely and then put into the 37 °C incubator for 30 min. After scaffold polymerization, fluorescence images were taken using the Axio Observer Z1 fluorescence microscope (Zeiss, Oberkochen, Germany).

### 2.5. Cell Culture of Human Umbilical Vein Endothelial Cells

Human Umbilical Vein Endothelial Cells (HUVECs) were purchased from PromoCell (Heidelberg, Germany) and cultured in endothelial cell growth medium 2 (EGM-2 kit, PromoCell, Heidelberg, Germany) with 1% amphotericin B and penicillin/streptomycin (Biochrom, Berlin, Germany) at 37 °C and 5% CO_2_. Cells in passages 5–7 were used for all experiments and medium change was performed three times per week.

### 2.6. Endothelial Tube Formation Assay

Tube formation assays were performed with HUVECs using a method adapted from Wang and co-authors [34]. Briefly, 100 μL/well of GeltrexTM LDEV-Free reduced growth factor basement membrane matrix (Invitrogen/Thermo Fisher Scientific, Waltham, MA, USA) was incubated in a 24-well plate for at least 30 min at 37 °C for matrix gel polymerization. HUVECs (5 × 10^4^) were then seeded onto GeltrexTM matrices and cultured for 8 h with EGM-2 medium containing VEGF protein (0.5 ng/mL, positive group), QK peptide (0.01 μM, 0.1 μM, 1 μM and 10 μM), E7-QK peptide (0.01 μM, 0.1 μM, 1 μM and 10 μM), or E7-QK-TAMRA peptide (1 μM). EGM-2 medium without VEGF protein served as negative control. After 8 h of cultivation at 37 °C, 1 μM calcein-AM dye (Invitrogen/Thermo Fisher Scientific, Waltham, MA, USA) was added to the plate and incubated for 20 min. Fluorescence images were captured from at least 3 wells per culture condition in a 1.25× magnification using the Axio Observer Z1 fluorescence microscope (Zeiss, Oberkochen, Germany). Network branches, meshes, and nodes were counted from the collected images using the tool Angiogenesis Analyzer of the ImageJ software, in order to quantify angiogenic network formation.

### 2.7. Spheroid Sprouting Assay in Collagen/Hydroxyapatite Scaffolds

Spheroid sprouting assay was adapted from the method previously described by Maracle and co-authors [35]. The principle of this assay is based on the sprout formation originating from aggregated and gel-embedded HUVECs. In brief, methocel solution was prepared by dissolving 3 g methylcellulose (Sigma, St. Louis, MO, USA) in 250 mL EGM-2 medium. (PromoCell, Heidelberg, Germany). HUVECs were then harvested. A total of 8000 HUVECs were added to each well of a 96 V-well polypropylene plate (Corning, Sigma, St. Louis, MO, USA) in 200 μL EGM-2 medium containing 20% methocel. Spheroids formed overnight at 37 °C. Then, spheroids were resuspended to 1 mL solution of prepared collagen/hydroxyapatite composites containing a 1 μM concentration of E7-QK or unmodified QK peptides. After incubation at 37 °C for 30 min for polymerization of the collagen/hydroxyapatite composites, 500 μL basal EGM-2 medium containing 10% FBS was added to the wells. Sprout formation by the HUVEC spheroids was detected after 48 h.

### 2.8. Fluorescence Staining

Collagen/hydroxyapatite peptide-functionalized scaffolds containing HUVEC spheroids were washed three times with PBS and fixed with 4% paraformaldehyde for 1 h. After cell permeabilization with PBS + 1% Triton-X100 (Sigma), cells were washed with PBS and stained with Alexa488-Phalloidin (10 μg/mL in bovine serum albumin, Sigma, St. Louis, MO, USA) and Hoechst 33342 (1 μg/mL, Promocell, Heidelberg, Germany) at room temperature for 1h. After three wash steps with PBS, images were taken using an Axio Observer Z1 fluorescence microscope (Zeiss, Oberkochen, Germany) in 10× and 20× magnifications. Spheroids were quantified using the length tool of the Axio Vision software of the Observer Z1 fluorescence microscope (Zeiss, Oberkochen, Germany) in order to measure the length of the sprouts and calculate the cumulative sprout length (CSL). Therefore, the lengths of all capillary-like sprouts originating from the central plain of an individual spheroid were added to one value. At least 10 spheroids per experimental group were analyzed.

### 2.9. RNA Isolation and Quantitative Gene Expression Analyses in HUVECs

RNA was isolated from HUVECs using the NucleoSpin RNA kit (Macherey-Nagel, Hoerd, France) according to the manufacturer’s guidelines. After isolation, RNA was quantified using a Nanodrop micro-volume spectrophotometer (Invitrogen/Thermo Fisher Scientific, Waltham, MA, USA). Then, cDNA synthesis was performed using the SuperScript™ VILO™ cDNA synthesis kit according to the instructions of the manufacturer (Invitrogen/Thermo Fisher Scientific, Waltham, MA, USA). To quantify mRNA expression levels, the real-time LightCycler System (Roche Diagnostics, Mannheim, Germany) was used. For the PCR reactions, DEPC-treated water, DNA Master SYBR Green I kit (Roche, Mannheim, Germany), and commercial primer kits (*FLT-1, KDR, HGF, MET, IL-8, HIF-1α, MMP-1, TIMP-1, IGFBP-1, IGFBP-2, VCAM-1*, and *ANGPT-1*) from Search LC (Heidelberg, Germany) were used for 40 amplification cycles of the target cDNA following the manufacturer’s instructions. The target gene transcript levels were normalized to those of the housekeeping gene *GAPDH*. X-fold induction values were calculated related to the corresponding control. All cDNA samples (from 3 independent experiments) for each experimental group (+VEGF (positive control), 1 µM E7-QK, 1 µM QK, −VEGF (negative control)) were analyzed in triplicates.

### 2.10. Statistical Analysis

The data of all measurements are expressed as means ± standard deviations (SD), except the data of sprouting assays, which are expressed as means ± standard error of means (SEM). All statistical analyses were carried out using the GraphPad Prism software (La Jolla, CA, USA). The two-tailed Student’s *t*-test or one-way analysis of variance (ANOVA) for repeated measurements followed by Tukey’s multiple comparisons tests were used. Values were considered significant with a *p*-value of <0.05.

## 3. Results

### 3.1. Binding Efficiency of Different VEGF Mimicry Peptides to HA

**Figure 1:**
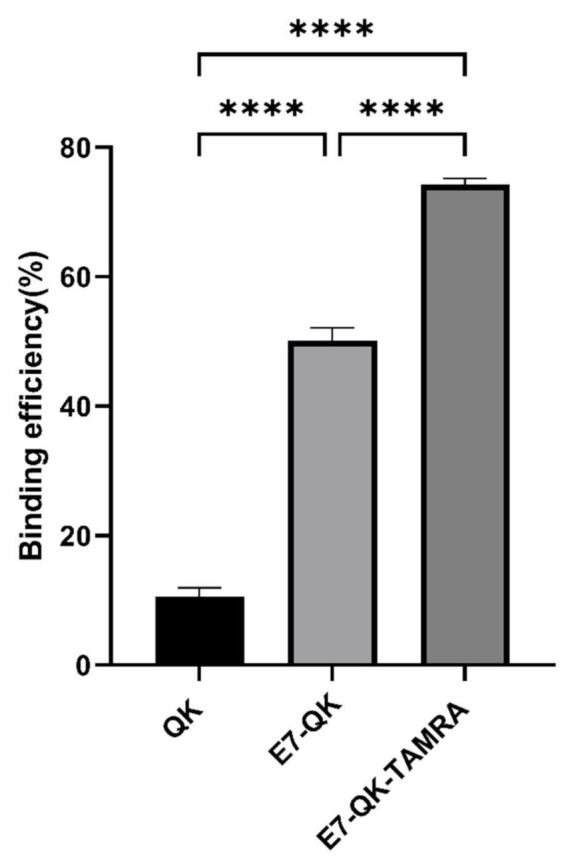
Binding efficiency of VEGF-mimicry peptides to HA particles. QK peptides (0.25 mM) with or without E7-tag and TAMRA-label were incubated with equilibrated HA particles for 2 h at RT. Biochemical measurements of peptide concentration were performed before and after incubation with HA particles. Binding efficiency (in percent) compared to the initially applied peptide concentration is illustrated in the diagram. **** *p* < 0.0001.

The binding efficiency of QK, E7-QK, and E7-QK-TAMRA peptides in equimolar amounts (0.25 mM) was quantified by measuring the amount of unbound peptide in supernatants after 2h of incubation with HA particles, using a BCA assay. As illustrated in Figure 1, 74.21 ± 0.98% of the E7-QK-TAMRA peptide, 50.12 ± 1.99% of E7-QK peptide, 10.55 ± 1.41% of QK peptide were detected to bind to HA particles. Differences between the three groups were highly significant (*n* = 7, *p* < 0.0001 among three groups).

### 3.2. Verification of QK and E7-QK Peptide Functionality Using In Vitro Angiogenesis Assays

**Figure 2:**
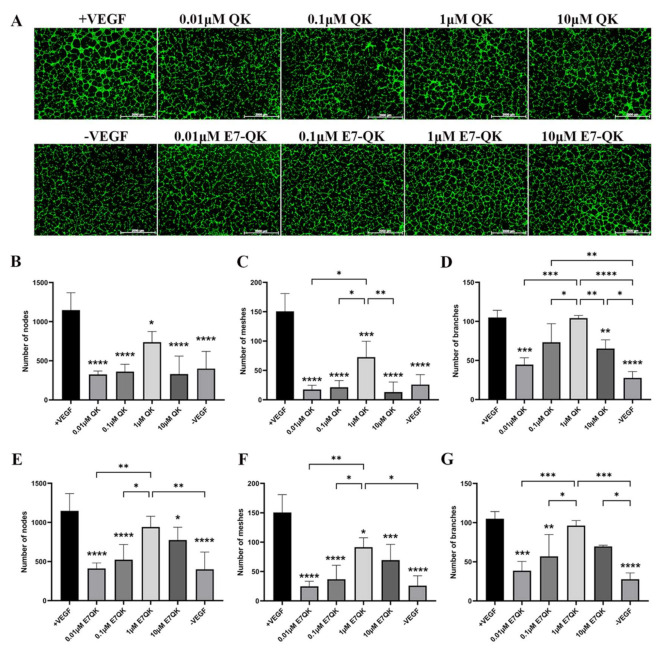
Tube formation of HUVECs incubated with different concentrations of VEGF and VEGF mimicry peptides (QK and E7-QK). HUVECs seeded on Geltrex matrix in medium containing different concentrations of QK or E7-QK peptide (0.01 µM, 0.1 µM, 1 µM, 10 µM), VEGF (0.5 ng/mL, positive control group), or without VEGF or peptides (negative control group). (**A**) Representative images (1.25× magnification, scale bar = 2000 µm) of tube formation were taken using fluorescent microscopy (Calcein AM) at 8 h after cell seeding. (**B**,**E**) Number of nodes, (**C**,**F**) number of meshes, and (**D**,**G**) number of branches were quantified with the ImageJ software. Values represent means ± SD from 3 independent experiments (*, **, ***, **** without bar indicates significant differences to the +VEGF group; * *p* < 0.05, ** *p* < 0.01, *** *p* < 0.001, **** *p* < 0.0001).

In order to evaluate the functionality of QK and E7-QK peptides, an endothelial tube formation assay was performed. After imaging, different amounts of tube-like structures were formed depending on the used angiogenic factor (peptides or VEGF). After quantification with Image J, we used three indicators to determine the angiogenic effects of the different peptides: number of nodes, number of meshes, and number of branches. As illustrated in Figure 2, cells in the VEGF positive group formed significantly more nodes (Figure 2B,E), meshes (Figure 2C,F), and branches (Figure 2D,G) than cells in all other groups. HUVECs treated with 1 μM E7-QK peptide and 1 μM QK peptide formed higher numbers of nodes, branches, and meshes compared to cells treated with peptides in other concentrations, or compared to cells from the negative group which were cultured in the absence of VEGF (-VEGF). Therefore, a 1 μM peptide concentration was chosen for all further experiments.

**Figure 3:**
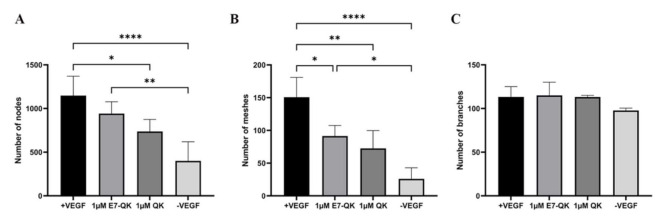
Quantification of tube formation by human umbilical vein endothelial cells in the presence of VEGF and VEGF mimicry peptides (1 µM). Quantification of fluorescent images was performed with the ImageJ software. Number of nodes (**A**), number of meshes (**B**), number of branches (**C**) formed by HUVECs in the presence of E7-QK (1 μM), and QK (1 μM) peptide, or in the presence of VEGF (0.5 ng/mL, positive control group), or without VEGF (negative control group) were compared. Values represent means ± SD from 3 independent experiments (* *p* < 0.05, ** *p* < 0.01, **** *p* < 0.0001).

The angiogenic effects of E7-QK and QK peptides on HUVECs at a concentration of 1 μM are illustrated in Figure 3. We found in the tendency higher numbers of nodes (3A) and meshes (3B) in the E7-QK group compared to the QK peptide group. However, differences between the two groups did not reach statistical significance. Further, we detected significantly higher numbers of nodes and meshes in the E7-QK cell group compared to the negative control group (-VEGF), whereas no significant differences were detected between the QK and the negative group.

### 3.3. Expression of Angiogenesis-Related Genes in HUVECs

**Figure 4:**
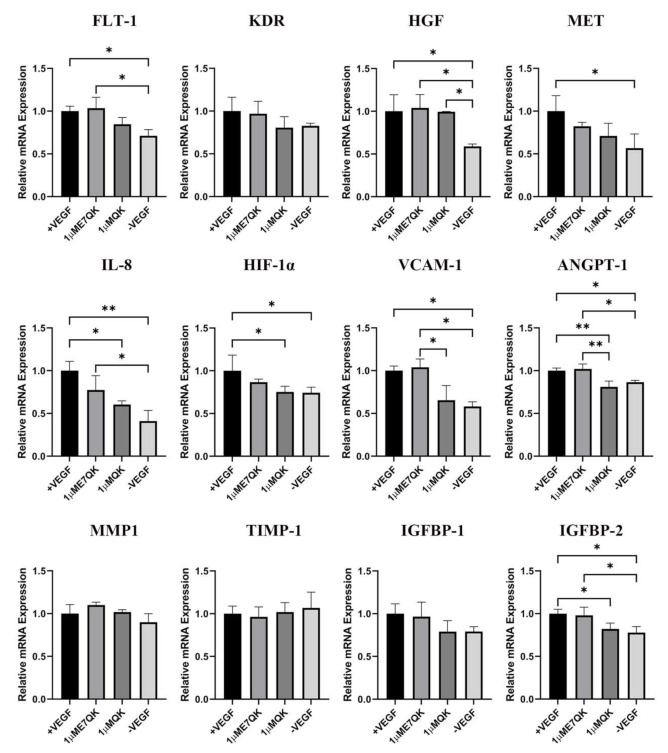
Expression of angiogenesis-related genes by HUVECs stimulated with QK and E7-QK peptide. Gene expression levels were quantified using a Light Cycler instrument, and ratios of mRNA copy numbers of the housekeeping gene (GAPDH) were calculated. Gene expression mean values ± SD in HUVECs from the 1 µM E7-QK peptide, QK peptide, VEGF (0.5 ng/mL, positive control group), or without VEGF (negative control group) groups were displayed as x-fold induction values relative to the VEGF positive group. Data were collected from three independent experiments (* *p* < 0.05; ** *p* < 0.01).

Further investigation of the biofunctionality of E7-QK and QK peptide includes the analysis of their effects on the angiogenic gene expression in HUVECs (Figure 4). HUVECs cultured in the presence of E7-QK peptide (1 μM) and VEGF (0.5 ng/mL, positive control group) showed higher gene expression levels of proangiogenic factors and their receptors compared to the negative control group. The expression of several genes reached significant differences, including the genes *FLT-1, HGF, MET, IL-8, HIF-1a, IGFBP-2, VCAM1*, and *ANGPT-1.* There were several genes that showed no significant differences, like *KDR, MMP1,* and *IGFBP-1*. In contrast, the expression of the anti-angiogenic gene *TIMP-1* showed lower levels in the E7-QK and +VEGF groups compared to those detected in the -VEGF group, however, without statistical significance.

### 3.4. Verification of the Functionality of E7-QK-TAMRA Compared to E7-QK Peptide Using In Vitro Angiogenesis Assays

**Figure 5:**
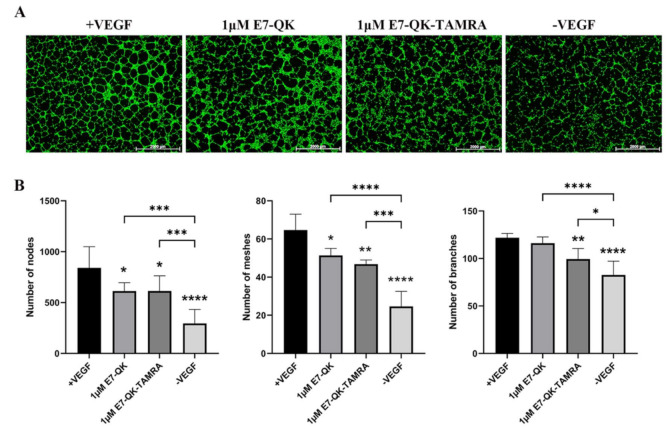
Tube formation by HUVECs in the presence of E7-QK-TAMRA peptide. HUVECs were seeded on Geltrex matrix in media containing 1 µM of E7-QK, E7-QK-TAMRA peptides, VEGF (0.5 ng/mL, positive control group), or without VEGF or peptides (negative control group). (**A**) Representative images (1.25× magnification, scale bar = 2000 µm) of HUVEC tube formation were taken by fluorescent microscopy (Calcein AM) at 8 h after cell seeding. (**B**) Numbers of nodes, meshes, and branches were analyzed with ImageJ. Values represent means ± SD from 3 independent experiments (*, **, ***, **** without bar indicates significant differences to the +VEGF group; * *p* < 0.05, ** *p* < 0.01, *** *p* < 0.001, **** *p* < 0.0001).

As shown in Figure 1, the binding efficiency of the modified E7-QK-TAMRA peptide to HA particles was significantly higher than the binding efficiency of the E7-QK peptide. In order to analyze the influence of TAMRA labeling of the E7-QK peptide on its angiogenic effects, we performed tube formation assays. As shown in Figure 5, E7-QK-TAMRA peptide induced significantly higher numbers of nodes and branches but no significantly different number of meshes, compared to the negative control group. The same number of nodes, significantly lower numbers of branches, and in the tendency lower numbers of meshes were formed by HUVECs in the E7-QK-TAMRA compared to the E7-QK cell group (Figure 5B). These results indicate that the additional modification with the TAMRA tag slightly reduced the angiogenic effect of the E7-QK peptide, despite the fact that the binding efficiency to HA particles was higher.

### 3.5. Detection and Distribution of VEGF-Mimicry Peptides within Collagen/Hydroxyapatite Composites

**Figure 6:**
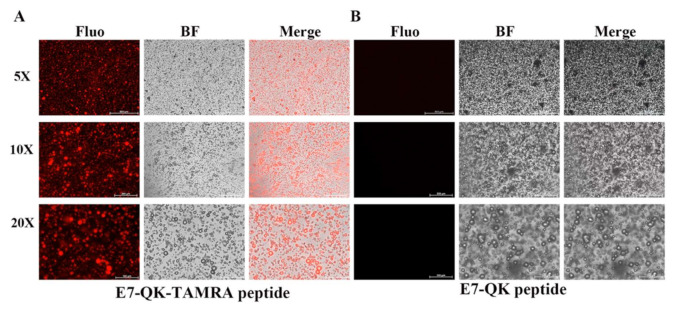
Detection of E7-QK and E7-QK-TAMRA peptide within coll/HA composites by fluorescence microscopy. E7-QK-TAMRA and E7-QK peptide bound to hydroxyapatite was incorporated into coll/HA composite scaffolds. Fluorescence images were captured with an Axio Observer Z1 fluorescence microscope, using a 5× (scale bar = 500 µm), 10× (scale bar = 200 µm), and 20× magnification (scale bar = 100 µm). (**A**) E7-QK-TAMRA peptide showed to be homogeneously distributed (red dots) within the coll/HA composites, whereas (**B**) Unlabeled E7-QK peptide was not visible within the scaffolds.

In order to detect and visualize the distribution of HA-bound VEGF-mimicry peptides within collagen/HA composites, we used a E7-QK peptide with a TAMRA red fluorochrome tag. After electrostatic binding of E7-QK-TAMRA peptide to HA, collagen/HA composites were generated. Visualization of the prepared scaffolds by fluorescence microscopy, showed a homogenous distribution of red dots within the prepared scaffolds (Figure 6), indicating that HA-bound peptide was distributed uniformly within the material.

### 3.6. HUVEC Spheroid Sprouting Assay within Collagen/Hydroxyapatite Composite Scaffolds

**Figure 7:**
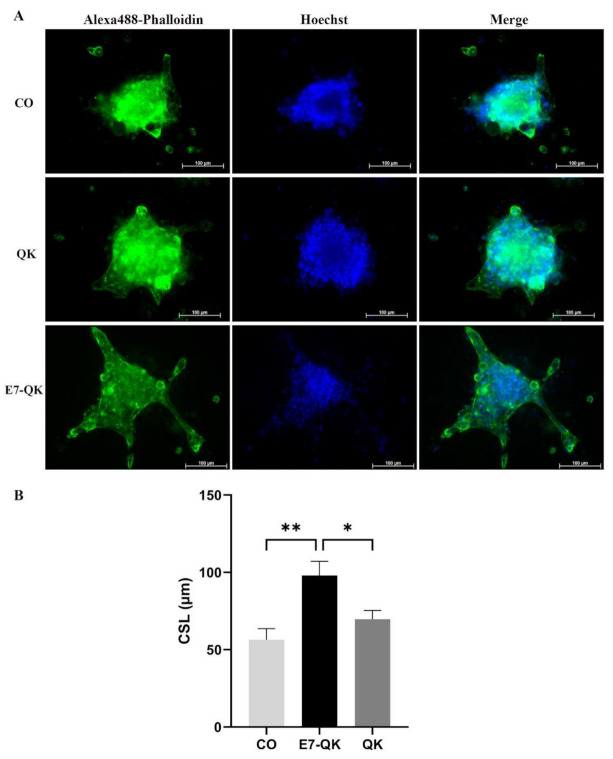
Sprout formation by 3D HUVEC spheroids cultured within coll/HA composites for 48 h. (**A**) Representative fluorescence images (Alexa488-Phalloidin staining) of HUVEC spheroids cultured within coll/HA composites without peptides (CO) or within coll/HA scaffolds comprising QK peptide (QK), and E7-QK peptide (E7-QK) in a 20× magnification (scale bar = 100 µm). (**B**) Quantification of cumulative sprouts length (CSL) was analyzed with the image analysis tool of the Axio Observer Z1 fluorescence microscope. Mean CSL was calculated for at least 10 randomly selected spheroids per experimental group. Values represent means ± SEM from 3 independent experiments (* *p* < 0.05; ** *p* < 0.01).

To evaluate the functionality of QK and E7-QK peptides also in the 3D-culture, sprouting assays with HUVEC spheroids were performed in collagen/hydroxyapatite (coll/HA) peptide-functionalized composite scaffolds. HUVEC spheroids were incorporated into coll/HA scaffolds containing E7-QK peptide, QK peptide or no peptides (CO) and sprout lengths were analyzed by fluorescence microscopy after 48 h (Figure 7A). By quantifying the cumulative sprout length (CSL), we detected a significantly higher CSL in the E7-QK peptide scaffold group (E7-QK), compared to the QK peptide scaffold group (QK) and the control group (CO). The CSL calculated for the QK group was in the tendency higher than that of the control group (CO), however differences did not reach significance (Figure 7B).

## 4. Discussion

Inorganic components such as hydroxyapatite or other calcium phosphates are frequently used in combination with organic components like collagen to mimic the natural bone composition for the fabrication of bone tissue engineering grafts [17,36,37]. In order to confer angiogenic properties to bone substitutes, the most promising strategy is targeting VEGF and its receptors [38]. VEGF is a key biomolecule in modulating angiogenesis, and it has been shown to promote osteogenesis as well, by modulating the VEGFR-1, 2 expression of osteoblasts [39,40,41]. The newly discovered and artificially synthesized VEGF-mimicry peptide QK was characterized in detail [24] 15 years ago. Since then, the QK peptide has been used in many studies to improve angiogenic properties of different implant materials. For instance, Chen and colleagues demonstrated that QK immobilized on titanium implants showed even antimicrobial effects and promoted vascularization and osseointegration in an in vivo animal model [42].

To enhance the binding of QK to HA, an E7 tag can be used, leading to a strong electrostatic binding of negatively charged glutamic acid residues to positively charged HA. By this modification, Pensa and colleagues achieved a 4–6-fold enrichment of QK peptide loaded onto two graft materials: anorganic bovine bone (ABB, BioOss, Geistlich, Baden-Baden, Germany) and synthetic HA [43].

In the current study, the functionality of QK and E7-QK peptides was evaluated in the 2D HUVEC culture and for the first time within a 3D collagen/hydroxyapatite composite scaffold. We were able to confirm previously published data from other research groups concerning similar levels of induced tube formation of 2D-cultured HUVECs by the addition of QK and E7-QK peptides, as detected after addition of the whole VEGF protein. As other authors measured peptide–mineral interactions by sophisticated techniques such as quartz crystal microbalance, atomic force microscopy, or surface plasmon resonance [44,45,46,47], we used a simple biochemical quantification method. By this approach, we could simply and reliably demonstrate that equimolar amounts of the unmodified and E7- and TAMRA-modified peptides led to significantly different binding affinities. In contrast to other studies, we provided evidence, for the first time, that the modification with the E7 tag does not interfere with the functionality of the QK peptide, but even improves levels of sprout formation by HUVEC spheroids in a 3D collagen/hydroxyapatite scaffold. We obtained these results notwithstanding the fact that we did not use equimolar amounts of QK and E7-QK peptides for the 3D experiment, but 200 µg of each peptide. This implicates that despite of a higher amount of QK (lower molecular weight) compared to the E7-QK peptide, its functionality was shown to be attenuated within the 3D scaffold, probably due to the weaker binding affinity of the QK peptide (5-fold lower) to hydroxyapatite.

In addition to tube formation assays, we analyzed the effects of E7-QK peptide on angiogenic gene expression in HUVECs. The analyzed genes play an important role during angiogenesis [48,49,50,51,52,53,54,55,56]. VEGF-A is the principle initiator of angiogenesis. After binding to its receptors VEGFR-1 (FLT-1) and VEGFR-2 (KDR), the angiogenic signaling is activated. As a response, endothelial cells release gelatinase (MMP-2) in the early stage of angiogenesis, in order to breakdown the basement membrane. For angiogenesis amplification and vascular stabilization, other factors like ANGPT-1, IL-8, IGFBP-1, VCAM-1, HIF-1α, and HGF come into play. Angiogenesis inhibiting factors like tissue inhibitors of metalloproteinases (TIMPs), endostatin, angiostatin, or thrombospondin suppress vascular growth. It has been shown that VEGF mimicry peptides interact with VEGF receptors, similarly to VEGF-A, inducing transcriptional changes in endothelial cells [57]. In our study, E7-QK peptide significantly induced gene expression levels of the receptor FLT-1 and the pro-angiogenic genes HGF, IL-8, IGFBP-2, VCAM-1, and ANGPT-1 in HUVECs compared to expression levels detected in the negative group (without VEGF or peptides). The induction of these proangiogenic genes proves, in addition to the performed tube formation assays, the biological functionality of the E7-QK peptide. In contrast, unmodified QK peptide only showed a significant increase of HGF gene expression compared to the negative group, clearly indicating a better biological functionality of the E7-modified peptide.

For drug delivery or tissue engineering applications, insoluble cross-linked hydrogels have been proposed. These materials allow immobilization and release of active biomolecules [58]. Our aim was to develop a 3D bone tissue engineering construct composed of both natural bone components type I collagen and hydroxyapatite. For this purpose, we combined a natural (bovine collagen) with a synthetic (hydroxyapatite) component and conferred angiogenic properties to the hybrid scaffold by the incorporation of E7-QK peptide. Using a TAMRA-labeled E7-QK peptide, we demonstrated high E7-QK peptide retention within the collagen/HA composites, as shown in Figure 6. In addition, we analyzed the biological activity of the E7-QK-TAMRA peptide using 2D tube formation assays. Compared with E7-QK peptide, tube formation of HUVECs incubated with E7-QK-TAMRA labeled peptide was slightly reduced, however, differences were not significant. This shows that angiogenic properties of E7-QK peptide are not inhibited by the TAMRA tag, which will enable experiments with simultaneous peptide detection in future studies.

For the investigation of the biological functionality of the E7-QK peptide within a 3D scaffold, we encapsulated HUVEC spheroids into collagen/HA composites containing these artificially synthesized biomolecules. The performed sprouting assays clearly demonstrated significantly higher sprout formation of HUVEC spheroids within collagen/HA composites containing E7-QK peptide, compared to the group of unmodified QK peptide and the negative control group (Figure 7). It seems that regardless of the cultivation approach—in 2D on a standard growth-factor reduced matrigel preparation composed of laminin I, type IV collagen, entactin, and heparan sulfate proteoglycans (information given by the company ThermoFisher Scientific), or in 3D within the type I collagen/hydroxyapatite constructs, HUVECs were able to be activated by the E7-QK peptide, either added to the culture as a soluble factor or electrostatically bound to hydroxyapatite. Taken together, in this study we provided evidence, that HA-bound E7-modified QK peptides show excellent functional properties and are able to induce sprout formation of endothelial cells within collagen/HA scaffolds.

## 5. Conclusions

In this study, we demonstrate that the VEGF-mimicry peptide QK modified with an E7-tag has a significantly higher binding affinity to hydroxyapatite through electrostatic attraction compared to unmodified QK-peptide. This non-covalent binding method provides a safe tool to add biological functionality to hydroxyapatite-based bone graft materials. Retention and enhanced biological activity of E7-QK peptide compared to the unmodified counterpart was demonstrated also within 3D Coll/HA composite scaffolds. Therefore, we present a promising approach for the development of a collagen/hydroxyapatite scaffold material exhibiting angiogenic properties for applications in the field of bone tissue engineering. Nevertheless, scaffold functionality in vivo has to be further investigated.

## Data Availability

Obtained data for this study are available from the corresponding author on reasonable request.
